# Echocardiographic Characteristics and Outcome in Patients With COVID-19 Infection and Underlying Cardiovascular Disease

**DOI:** 10.3389/fcvm.2021.642973

**Published:** 2021-03-16

**Authors:** Yuman Li, Lingyun Fang, Shuangshuang Zhu, Yuji Xie, Bin Wang, Lin He, Danqing Zhang, Yongxing Zhang, Hongliang Yuan, Chun Wu, He Li, Wei Sun, Yanting Zhang, Meng Li, Li Cui, Yu Cai, Jing Wang, Yali Yang, Qing Lv, Li Zhang, Amer M. Johri, Mingxing Xie

**Affiliations:** ^1^Department of Ultrasound, Tongji Medical College, Union Hospital, Huazhong University of Science and Technology, Wuhan, China; ^2^Clinical Research Center for Medical Imaging in Hubei Province, Wuhan, China; ^3^Hubei Province Key Laboratory of Molecular Imaging, Wuhan, China; ^4^Department of Medicine, Queen's University, Kingston, ON, Canada

**Keywords:** COVID-19, cardiovascular disease, echocardiography, cardiac injury, cardiac function

## Abstract

**Background:** The cardiac manifestations of coronavirus disease 2019 (COVID-19) patients with cardiovascular disease (CVD) remain unclear. We aimed to investigate the prognostic value of echocardiographic parameters in patients with COVID-19 infection and underlying CVD.

**Methods:** One hundred fifty-seven consecutive hospitalized COVID-19 patients were enrolled. The left ventricular (LV) and right ventricular (RV) structure and function were assessed using bedside echocardiography.

**Results:** Eighty-nine of the 157 patients (56.7%) had underlying CVD. Compared with patients without CVD, those with CVD had a higher mortality (22.5 vs. 4.4%, *p* = 0.002) and experienced more clinical events including acute respiratory distress syndrome, acute heart injury, or deep vein thrombosis. CVD patients presented with poorer LV diastolic and RV systolic function compared to those without CVD. RV dysfunction (30.3%) was the most frequent, followed by LV diastolic dysfunction (9.0%) and LV systolic dysfunction (5.6%) in CVD patients. CVD patients with high-sensitivity troponin I (hs-TNI) elevation or requiring mechanical ventilation therapy demonstrated worsening RV function compared with those with normal hs-TNI or non-intubated patients, whereas LV systolic or diastolic function was similar. Impaired RV function was associated with elevated hs-TNI level. RV function and elevated hs-TNI level were independent predictors of higher mortality in COVID-19 patients with CVD.

**Conclusions:** Patients with COVID-19 infection and underlying CVD displayed impaired LV diastolic and RV function, whereas LV systolic function was normal in most patients. Importantly, RV function parameters are predictive of higher mortality.

## Introduction

Coronavirus disease 2019 (COVID-19) caused by the severe acute respiratory syndrome coronavirus 2 (SARS-CoV-2) has become a global pandemic causing an escalating number of cases and fatalities worldwide. A large proportion of COVID-19 patients have comorbidities, with cardiovascular disease (CVD) being the most frequent. It was present in approximately 30–48% of patients (1–3). Patients with CVD are more likely to be infected with SARS-CoV-2 and to develop severe cases. In SARS, the presence of comorbidity increased the risk of death 12-fold (4). Therefore, COVID-19 patients with underlying CVD may suffer from a higher risk of mortality after SARS-CoV-2 infection (3, 5). A recent study revealed that hospitalized COVID-19 patients with concomitant cardiac disease have an exceptionally poor prognosis compared with those without cardiac disease (6). Nevertheless, the detailed features of cardiac function were not yet established in the aforementioned study. In clinical practice, echocardiography is the first-line imaging modality in cardiac assessment and is an indispensable bedside tool, allowing non-invasive quantification of heart performance in COVID-19 patients in isolated wards (7). Currently, there are limited data regarding the cardiac manifestations of COVID-19 patients with CVD. Therefore, we aimed to investigate the echocardiographic characteristics and explore the prognostic value of echocardiographic parameters in COVID-19 patients with CVD.

## Methods

### Study Population

This observational study was performed at the west branch of Union Hospital, Tongji Medical College, Huazhong University of Science and Technology of Wuhan, China, which was a designated hospital to treat patients with COVID-19. We enrolled a total of 157 consecutive adult patients who were confirmed to have COVID-19 infection according to the WHO interim guidance from February 12, 2020 to March 16, 2020 (8). Bedside echocardiography was performed in all patients from three wards managed by the investigators for evaluation of cardiac function. The study was approved by Union Hospital Tongji Medical College, Huazhong University of Science and Technology Ethics Committee (KY-2020-02.06). Written informed consent was waived for all participants with emerging infectious diseases as per the Ethics Committee.

### Data Collection and Definitions

Epidemiological, medical history, comorbidities, laboratory, treatment, and outcomes data were collected from electronic medical records. The data were analyzed by a trained team of physicians. The timing of laboratory measurements was within 3 days of echocardiographic examination with a mean interval of 1 day [interquartile range (IQR), 1–2]. The median time from admission to echocardiographic examination was 7 days (IQR, 3–11). Clinical outcomes (death or discharge) were monitored through to April 7th, 2020.

Underlying CVD included a history of hypertension, coronary artery disease, heart failure, cardiomyopathy, and arrhythmia. Acute cardiac injury was defined as serum levels of cardiac high-sensitivity troponin I (hs-TNI) above the 99th percentile upper reference limit.

### Echocardiography

Bedside echocardiography examinations were performed with an EPIQ 7C machine (Philips Medical Systems, Andover, MA, USA) at the designated COVID-19 isolation wards or intensive care units (ICU). Two-dimensional and Doppler echocardiography were performed in standard views according to the American Society of Echocardiography (ASE) guidelines (9). All scans were conducted by trained individuals in full personal protective equipment (PPE) (B.W., L.H., D.Z., Y.Z., H.Y., C.W., and H.L.). Personal protection at the time of echocardiographic assessment included wearing protective clothing, double gloving, shoe covers, head covers, N95 respirator masks, goggles, face shields. All images were stored in the ultrasound machine. At the end of the day, images were copied to hard disk and saved in Digital Imaging for subsequent offline analysis to reduce exposure contamination. Echocardiographic image readers (S.Z., W.S., Y.C., and L.C.) were blinded to epidemiological, clinical, laboratory, treatment, and outcomes findings.

### Left Heart Assessment

Left ventricular (LV) ejection fraction (LVEF) and volumes were calculated using Simpson's biplane method. LV mass was calculated according to Devereux's formula. LV diastolic function was estimated using the ratio of early transmitral flow velocity (E) to the late transmitral flow velocity (A) and the ratio of transmitral E to the early diastolic LV septal tissue velocity (e′). LV systolic dysfunction was defined as a LVEF <50%, and LV diastolic dysfunction was determined according to the published guideline of the American Society of Echocardiography (ASE) and the European Association of Cardiovascular Imaging (EACVI) (10).

### Right Heart Evaluation

**Figure 1:**
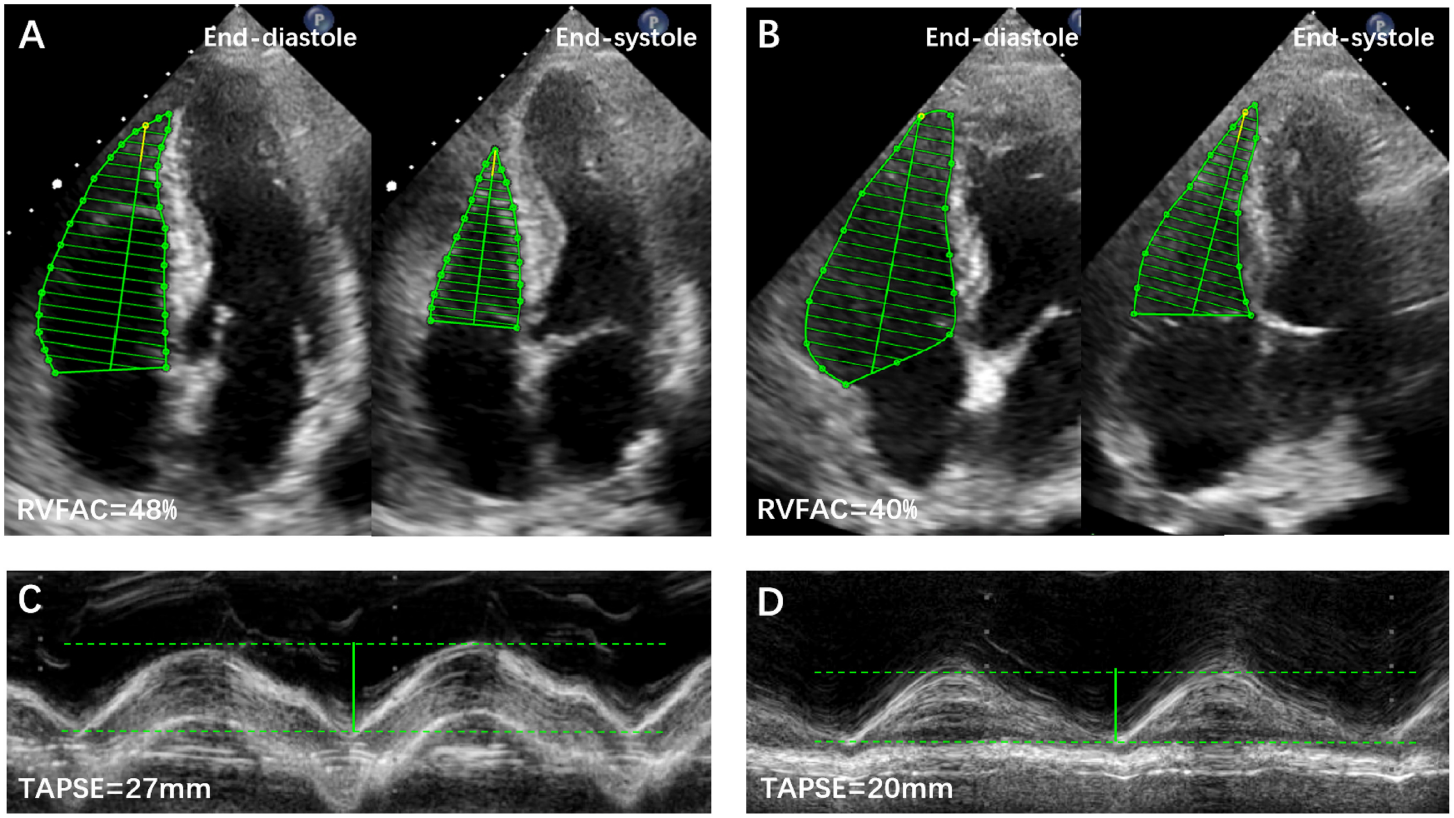
Representative examples of RVFAC and TAPSE measurements from COVID-19 Patients without and with CVD. **(A)** RVFAC in COVID-19 patient without CVD. **(B)** RVFAC in COVID-19 patient with CVD. **(C)** TAPSE in COVID-19 patient without CVD. **(D)** TAPSE in COVID-19 patient with CVD. CVD, cardiovascular disease; RVFAC, right ventricular fractional area change; TAPSE, tricuspid annular plane systolic excursion.

RV function was assessed by tricuspid annular plane systolic excursion (TAPSE), fractional area change (FAC), peak systolic velocity (S′) of the tricuspid lateral annulus, and myocardial performance index (MPI) (9). RV dysfunction was defined as the aforementioned parameters measured to be lower than the published reference values (9). Representative examples of RVFAC and TAPSE measurements from COVID-19 patients without and with CVD are shown in Figure 1. The degree of tricuspid regurgitation (TR) was defined as moderate, moderate to severe, or severe TR. Pulmonary artery systolic pressure (PASP) was estimated according to published guidelines (9).

### Statistical Analysis

Continuous numeric variables are expressed as mean ± SD or medians (interquartile range), and categorical variables are expressed as frequency (percentage). Continuous variables were compared using a two-sample *t*-test or Mann–Whitney test. Categorical variables were compared using the χ^2^-test or Fisher's exact test. Correlations between echocardiographic and biomarker parameters were examined using Spearman's correlation coefficient. Receiver operator characteristic (ROC) curves were used to evaluate the optimal cutoff value (maximum Youden index) of LV and RV function parameters for detecting poor outcome. Survival curves were plotted using the Kaplan–Meier analysis and compared using the log-rank test. To investigate the risk factors associated with in-hospital death, univariate and multivariate Cox regression models were used. All potential explanatory variables entered into univariate analyses, including age, sex, laboratory findings, LV and RV echocardiographic parameters, and comorbidities. Variables with *p* < 0.05 in univariate Cox proportional hazard regression were included in the multivariate model. To assess the additional prognostic value of echocardiographic parameters over other clinical variables, likelihood ratio tests were performed, and Akaike information criterion (AIC) and Harrell's C statistic were calculated. All statistical analyses were performed using SPSS version 24.0 (SPSS Inc., Chicago, Illinois) and R version 3.6.3 (R Foundation for Statistical Computing, Vienna, Austria). Statistical charts were generated using Prism 7 (GraphPad) and Minitab (Version 18). A two-sided *p* < 0.05 was considered as statistically significant.

## Results

### Clinical and Echocardiographic Characteristics in Patients With COVID-19 and CVD

Clinical characteristics of patients with COVID-19 with and without CVD are shown in Table 1. Among the 157 hospitalized patients with COVID-19, 134 (85.4%) patients were discharged and 23 (14.6%) patients died. The mean age was 62 ± 13 years, and 79 (50.3%) were men. Eighty-nine (56.7%) patients had underlying CVD. Among the CVD patients, hypertension, coronary artery disease, heart failure, and arrhythmia were present in 78.7, 29.2, 4.5, and 6.7% of the patients, respectively. Compared with patients without CVD, those with pre-existing CVD were older, and a higher proportion were men (42.7% female). Patients with underlying CVD were more likely to have a higher systolic arterial pressure, lower level of lymphocyte count and partial pressure of arterial oxygen to percentage of inspired oxygen ratio (PaO_2_: FIO_2_), higher levels of serum hs-TNI and B-type natriuretic peptide (BNP), more treatment with antibiotic, high-flow oxygen and mechanical ventilation, higher rate of ICU admissions, and higher incidence of acute respiratory distress syndrome (ARDS), acute heart injury, and deep vein thrombosis (DVT). Mortality was significantly higher in CVD compared with non-CVD patients (22.5 vs. 4.4%, *p* = 0.002).

**Table 1:** Clinical characteristics of patients with COVID-19 infection with and without cardiovascular disease.

**Variables**	**All patients**	**With CVD**	**Without CVD**	***P*-value**
	**(*n* = 157)**	**(*n* = 89)**	**(*n* = 68)**	
**Clinical characteristic**				
Age, years	62 ± 13	66 ± 11	58 ± 14	<0.001
Male, *n* (%)	79 (50.3%)	51 (57.3%)	28 (41.2%)	0.045
Body mass index, kg/m^2^	24.1 ± 3.1	24.0 ± 3.0	24.3 ± 3.1	0.445
Heart rate, beats/min	90 ± 17	89 ± 16	92 ± 17	0.164
Respiratory rate, breaths/min	25 ± 6	25 ± 6	25 ± 6	0.780
Systolic arterial pressure, mm Hg	133 ± 81	138 ± 17	126 ± 17	<0.001
Diastolic arterial pressure, mm Hg	81 ± 12	82 ± 13	80 ± 10	0.096
Smoker, *n* (%)	17 (10.8%)	11 (12.4%)	6 (8.8%)	0.480
**Comorbidities**				
Hypertension, *n* (%)	70 (44.6%)	70 (78.7%)	0 (0%)	<0.001
Diabetes, *n* (%)	23 (14.6%)	17 (19.1%)	6 (8.8%)	0.071
Obesity, *n* (%)	24 (15.3%)	15 (16.9%)	9 (13.2%)	0.532
COPD, *n* (%)	9 (5.7%)	6 (6.7%)	3 (4.4%)	0.534
Coronary artery disease, *n* (%)	26 (16.6%)	26 (29.2%)	0 (0%)	<0.001
Heart failure, *n* (%)	4 (2.5%)	4 (4.5%)	0 (0%)	0.077
Arrhythmia, *n* (%)	6 (3.8%)	6 (6.7%)	0 (0%)	0.029
Chronic kidney disease, *n* (%)	3 (1.9%)	2 (2.2%)	1 (1.5%)	0.725
Chronic liver disease, *n* (%)	6 (3.8%)	2 (2.2%)	4 (5.8%)	0.234
Malignancy, *n* (%)	11 (7.0%)	3 (3.4%)	8 (11.8%)	0.041
**Laboratory findings**				
Lymphocyte count, ×10^9^/L	1.0 (0.6, 1.4)	0.9 (0.5, 1.2)	1.0 (0.7, 1.5)	0.012
D-dimer, mg/L	1.1 (0.4, 2.7)	1.5 (0.4, 2.4)	1.0 (0.5, 4.2)	0.295
PT, s	13.5 (12.5, 15.0)	13.4 (12.6, 15.2)	13.7 (12.5, 14.5)	0.99
APTT, s	37.4 (33.3, 44.6)	38.0 (33.1, 45.6)	37.0 (33.7, 42.2)	0.555
CK-MB, U/L	11 (8, 18)	12 (8, 25)	10 (8, 13)	0.05
hs-TNI, ng/L	4.8 (2.2, 31.2)	10.6 (3.3, 53.7)	2.7 (1.7, 7)	0.043
BNP, pg/ml	79.1 (35.7, 163.9)	85.3 (34.6, 162.5)	57.9 (38.7, 153.2)	0.049
CRP, mg/L	26.5 (3.7, 67.6)	27.5 (7.1, 75.4)	25.3 (2.8, 63.2)	0.44
PCT, ng/ml	0.08 (0.05, 0.20)	0.10 (0.05, 0.20)	0.07 (0.05, 0.21)	0.244
IL-6, pg/ml	5.2 (2.4, 20.7)	8.9 (3.5, 21.6)	4.6 (2.5, 21.7)	0.269
PaO_2_:FIO_2_, mmHg	232.0 (151.0, 268.97)	212.1 (140.6, 241.5)	254.0 (212.1, 330.5)	0.016
**Treatments**				
Antiviral therapy, *n* (%)	150 (95.5%)	86 (96.6%)	64 (94.1%)	0.45
Antibiotic therapy, *n* (%)	119 (75.8%)	73 (82.0%)	46 (67.6%)	0.037
Glucocorticoid therapy, *n* (%)	65 (41.4%)	36 (40.4%)	29 (42.6%)	0.782
Intravenous immune globulin, *n* (%)	56 (35.9%)	37 (41.6%)	19 (27.9%)	0.089
Anticoagulant therapy, *n* (%)	81 (51.6%)	52 (58.4%)	29 (42.6%)	0.05
Diuretics, *n* (%)	39 (24.8%)	32 (36.0%)	7 (10.3%)	<0.001
Beta-blockers, *n* (%)	33 (21.0%)	28 (31.5%)	5 (7.4%)	<0.001
Calcium channel blockers, *n* (%)	48 (30.6%)	43 (48.3%)	5 (7.4%)	<0.001
ACE-I/ARB, *n* (%)	17 (10.8%)	15 (16.9%)	2 (2.9%)	0.005
Oxygen therapy, *n* (%)	139 (88.5%)	83 (93.3%)	56 (82.3%)	0.034
High-flow oxygen, *n* (%)	90 (57.3%)	61 (68.5%)	29 (42.6%)	0.001
Mechanical ventilation, *n* (%)	37 (23.6%)	27 (30.3%)	10 (14.7%)	0.022
IMV, *n* (%)	26 (16.6%)	19 (21.3%)	7 (10.3%)	0.065
NIMV, *n* (%)	11 (7.0%)	8 (9.0%)	3 (4.4%)	0.266
ICU admission, *n* (%)	27 (17.2%)	20 (22.5%)	7 (10.3%)	0.045
**Complications**				
Acute kidney injury, *n* (%)	20 (12.8%)	12 (13.5%)	8 (11.8%)	0.775
ARDS, *n* (%)	64 (40.8%)	47 (52.8%)	17 (25.0%)	<0.001
Acute heart injury, *n* (%)	48 (20.6%)	35 (39.3%)	13 (19.1%)	0.006
Coagulation dysfunction, *n* (%)	29 (18.5%)	19 (21.3%)	10 (14.7%)	0.288
DVT, *n* (%)	63 (40.1%)	42 (47.2%)	21 (30.9%)	0.039
Shock, *n* (%)	1 (0.6%)	1 (1.1%)	0 (0%)	0.567
**Prognosis**				
Discharge, *n* (%)	134 (85.4%)	69 (77.5%)	65 (95.6%)	0.002
Death, *n* (%)	23 (14.6%)	20 (22.5%)	3 (4.4%)	0.002

*Values are mean ± SD, n (%), median (interquartile range)*.

*ACE-I, angiotensin-converting enzyme inhibitors; APTT, activated partial thromboplastin time; ARB, angiotensin II receptor blockers; ARDS, acute respiratory distress syndrome; BNP, B-type natriuretic peptide; CK-MB, creatine kinase muscle–brain; COVID-19, coronavirus disease 2019; COPD, chronic obstructive pulmonary disease; CRP, C-reactive protein; CVD, cardiovascular disease; DVT, deep vein thrombosis; FIO_2_, fraction of inspiration oxygen; HF, heart failure; hs-TNI, high-sensitivity troponin I; ICU, intensive care unit; IL-6, interleukin-6; IMV, invasive mechanical ventilation; NIMV, non-invasive mechanical ventilation; PCT, procalcitonin; PT, prothrombin time; PaO_2_, partial pressure of oxygen*.

Echocardiographic characteristics of COVID-19 patients with and without CVD are depicted in Table 2. Compared with patients without CVD, those with CVD had impaired LV diastolic and RV function and a higher PASP. No differences were identified in LV wall thickness and mass, LV volumes, LVEF, and mitral regurgitation (MR) or TR severity. The most frequent cardiac abnormality in CVD patients was RV dysfunction (27/89, 30.3%), followed by LV diastolic dysfunction (8/89, 9.0%) and LV systolic dysfunction (5/89, 5.6%).

**Table 2:** Echocardiographic characteristics of patients with COVID-19 with and without cardiovascular disease.

**Variables**	**All patients**	**With CVD**	**Without CVD**	***P*-value**
	**(*n* = 157)**	**(*n* = 89)**	**(*n* = 68)**	
**Left heart**				
LA dimension, mm	35.4 ± 5.5	36.7 ± 5.9	33.3 ± 4.3	<0.001
LV dimension, mm	45.7 ± 5.1	45.7 ± 5.0	45.7 ± 5.2	0.967
IVS, mm	9.6 ± 1.2	9.7 ± 1.3	9.5 ± 1.0	0.125
PW, mm	9.1 ± 1.3	9.2 ± 1.4	8.9 ± 1.2	0.291
LVMI, g/m^2^	86.9 ± 21.0	88.4 ± 23.4	84.7 ± 16.9	0.331
Mitral DT, ms	203 ± 55	206 ± 53	200 ± 58	0.561
Mitral E/A	0.91 ± 0.36	0.88 ± 0.33	0.96 ± 0.39	0.473
Mitral E/e′	9.2 ± 3.2	9.7 ± 3.4	8.5 ± 2.8	0.043
LVEDVI, ml/m^2^	51.3 (43.8, 62.5)	53.5 (43.0, 64.7)	50.7 (44.0, 58.0)	0.173
LVESVI, ml/m^2^	19.3 (15.6, 25.7)	21.7 (15.6, 28.1)	18.6 (15.6, 23.8)	0.085
LVEF, %	63.4 ± 7.0	62.5 ± 8.3	64.7 ± 4.7	0.063
Moderate-severe MR, *n* (%)	6 (3.9%)	5 (5.6%)	1 (1.5%)	0.179
**Right heart**				
RA dimension, mm	35.8 ± 5.0	36.6 ± 5.3	34.9 ± 4.4	0.042
RV dimension, mm	34.6 ± 5.5	34.9 ± 5.6	34.2 ± 5.3	0.390
Tricuspid E/A	0.96 ± 0.29	0.92 ± 0.29	1.0 ± 0.29	0.134
Tricuspid E/e′	5.5 ± 1.8	5.7 ± 1.7	5.2 ± 2.0	0.577
TAPSE, mm	22.2 ± 3.8	21.5 ± 3.7	23.2 ± 3.9	0.007
RV FAC, %	47.5 ± 6.8	46.0 ± 5.3	49.3 ± 7.3	0.009
S′, cm/s	13.5 ± 3.2	13.4 ± 3.1	13.5 ± 3.4	0.946
RV MPI	0.46 ± 0.14	0.48 ± 0.16	0.43 ± 0.10	0.011
Moderate-severe TR, *n* (%)	6 (3.9%)	5 (5.6%)	1 (1.5%)	0.179
PASP, mmHg	32 (24, 47)	42 (27, 50)	28 (24, 39)	0.033

*Values are mean ± SD, n (%), median (interquartile range). COVID-19, coronavirus disease 2019; CVD, cardiovascular disease; DT, deceleration time; IVS, interventricular septum; LA, left atrium; LV, left ventricular; LVEDVI, left ventricular end diastolic volume index; LVESVI, left ventricular end systolic volume index; LVEF, left ventricular ejection fraction; LVM, left ventricular mass; MR, mitral regurgitation; RA, right atrium; RV, right ventricular; TAPSE, tricuspid annular plane systolic excursion; RV FAC, RV fractional area change; RV MPI, RV myocardial performance index; TR, tricuspid regurgitation; PASP, pulmonary artery systolic pressure; PW, posterior wall of left ventricle*.

At the time of echocardiographic examination, 27 (30%) COVID-19 patients with CVD were treated with mechanical ventilation. These mechanically ventilated patients had decreased TAPSE and RVFAC and higher PASP, suggesting impaired RV function (Supplementary Table 1). In contrast, LV systolic or diastolic function was not different between patients with and without mechanical ventilation therapy.

### Biomarker Levels and Echocardiography in COVID-19 Patients With CVD

Echocardiographic findings in COVID-19 patients with CVD stratified by hs-TNI level are shown in Table 3. Patients with high hs-TNI levels had worse RV function, as evidenced by lower TAPSE and RVFAC, and higher MPI, whereas LV diastolic or systolic function did not differ between patients with and without hs-TNI elevation. Correlations of hs-TNI level with LV and RV parameters are displayed in Supplementary Table 2. hs-TNI level negatively correlated with tricuspid E/A, TAPSE, and RVFAC and positively correlated with LA and right heart dimension, mitral E/e′, and RVMPI.

**Table 3:** Clinical and echocardiographic characteristics of COVID-19 patients with CVD stratified by hs-TNI level.

**Variables**	**Normal hs-TNI (*N* = 58)**	**Elevated hs-TNI (*N* = 31)**	***P-*value**
Age, years	65 ± 11	68 ± 10	0.185
Male, *n* (%)	27 (46.6%)	24 (77.4%)	0.003
Body mass index, kg/m2	23.8 ± 2.9	24.2 ± 3.3	0.629
Heart rate, beats/min	88 ± 17	91 ± 15	0.426
Respiratory rate, times/min	25 ± 6	25 ± 7	0.637
Systolic arterial pressure, mm Hg	139 ± 18	134 ± 16	0.216
Diastolic arterial pressure, mm Hg	83 ± 13	80 ± 13	0.236
CK-MB, U/L	10 (7, 14)	22 (13, 33)	0.072
BNP, pg/ml	53.2 (26.6, 111.8)	138.6 (86.9, 279)	0.062
CRP, mg/L	16.2 (4.2, 16.2)	62.9 (22.7, 124.5)	0.002
PCT, ng/ml	0.07 (0.05, 0.11)	0.21 (0.08, 0.40)	0.003
IL-6, pg/ml	4.5 (3.0, 14.8)	14 (10.5, 71)	0.126
D-dimer, mg/L	0.9 (0.3, 2.1)	1.7 (0.9, 3.0)	0.262
**Left heart**			
LA dimension, mm	35.7 ± 5.2	38.6 ± 6.5	0.029
LV dimension, mm	45.7 ± 4.9	45.8 ± 5.3	0.913
IVS, mm	9.8 ± 1.2	9.7 ± 1.5	0.653
PW, mm	9.0 ± 1.4	9.4 ± 1.3	0.206
LVMI, g/m^2^	87.4 ± 20.5	90.2 ± 28.3	0.628
Mitral E/A	0.82 ± 0.29	0.97 ± 0.38	0.050
Mitral E/e′	9.1 ± 3.0	10.5 ± 3.9	0.084
LVEDVI, ml/m^2^	53.0 (42.1, 68.8)	53.5 (45.5, 62.5)	0.079
LVESVI, ml/m^2^	21.6 (16.0, 31.1)	23.4 (15.0, 25.3)	0.061
LVEF, %	61.6 ± 8.9	64.2 ± 6.8	0.203
**Right heart**			
RA dimension, mm	35.6 ± 4.6	38.1 ± 6.1	0.038
RV dimension, mm	34.2 ± 5.3	36.1 ± 6.0	0.134
Tricuspid E/A	0.92 ± 0.30	0.92 ± 0.30	0.985
Tricuspid E/e′	4.8 ± 2.2	5.5 ± 2.4	0.147
TAPSE, mm	22.2 ± 3.7	20.1 ± 3.3	0.013
RVFAC, %	47.2 ± 6.1	43.6 ± 5.0	0.020
S′, cm/s	13.5 ± 3.3	13.4 ± 2.8	0.855
RV MPI	0.45 ± 0.14	0.54 ± 0.17	0.018
PASP, mmHg	32 (26, 40)	47 (34, 56)	0.009

*Data are mean ± SD, n (%), median (IQR). hs-TNI elevation was defined as higher than 26.5 ng/L*.

*SD, standard deviation; IQR, interquartile range. BNP, B-type natriuretic peptide; CK-MB, creatine kinase muscle-brain; CRP, C-reactive protein; hs-TNI, high-sensitivity troponin I; IL-6, interleukin-6; PCT, procalcitonin; COVID-19, coronavirus disease 2019; CVD, cardiovascular disease; IVS, interventricular septum; LA, left atrium; LV, left ventricular; LVEDVI, left ventricular end diastolic volume index; LVEF, left ventricular ejection fraction; LVESVI, left ventricular end systolic volume index; LVM, left ventricular mass; MPI, myocardial performance index; PW, posterior wall of left ventricle; RA, right atrium; RV, right ventricular; RVFAC, right ventricular fractional area change; RV MPI, RV myocardial performance index; TAPSE, tricuspid annular plane systolic excursion; PASP, pulmonary artery systolic pressure*.

### Clinical and Echocardiographic Characteristics of Survivors and Non-survivors Among CVD Patients

Clinical characteristics of survivors and non-survivors among CVD patients are presented in Supplementary Table 3. Compared with CVD patients who were alive, those who died were more likely to have been male and have a lower lymphocyte count, higher levels of biomarkers, more likely to be treated with glucocorticoids, intravenous immune globulins, anticoagulants, diuretics, high-flow oxygen, and mechanical ventilation, and had a higher rate of admission to the ICU. Among the complications, acute kidney injury, acute heart injury, ARDS, coagulation dysfunction, and DVT were more common in non-survivors than survivor.

Echocardiographic characteristics of survivors and non-survivors among CVD patients are depicted in Table 4. Compared with survivors, non-survivors had enlarged left atrial size, lower RV function, and higher PASP, while LV systolic or diastolic function was similar between survivors and non-survivors. Of these non-survivors, 12/20 (60%) patients had RV dysfunction, while only 1/20 (5%) had LV diastolic dysfunction.

**Table 4:** Echocardiographic characteristics of COVID-19 patients with CVD stratified by vital status.

	**With CVD (*n* = 89)**	**Survivors (*n* = 69)**	**Non-survivors (*n* = 20)**	***P-*value**
**Left heart**				
LA dimension, mm	36.7 ± 5.9	36.2 ± 6.2	38.3 ± 4.3	0.035
LV dimension, mm	45.7 ± 5.0	46.0 ± 5.1	44.9 ± 4.6	0.460
IVS, mm	9.7 ± 1.3	9.9 ± 1.3	9.4 ± 1.3	0.230
PW, mm	9.2 ± 1.4	9.1 ± 1.4	9.3 ± 1.2	0.853
LVMI, g/m^2^	88.4 ± 23.4	90.8 ± 24.6	80.4 ± 17.4	0.141
Mitral DT	206 ± 53	210 ± 54	187 ± 45	0.142
Mitral E/A	0.88 ± 0.33	0.80 (0.67, 1.00)	0.72 (0.67, 0.80)	0.110
Mitral E/e′	9.7 ± 3.4	9.7 ± 3.5	9.7 ± 3.0	0.713
LVEDVI, ml/m^2^	53.5 (43.0, 64.7)	52.4 (40.3, 67.2)	53.6 (46.4, 59.4)	0.257
LVESVI, ml/m^2^	21.7 (15.6, 28.1)	20.9 (15.8, 28.1)	23.4 (14.6, 29.8)	0.505
LVEF, %	62.5 ± 8.3	61.7 ± 8.6	65.4 ± 6.6	0.083
Moderate-severe MR, *n* (%)	5 (5.6%)	2 (2.8%)	3 (15%)	0.073
**Right heart**				
RA dimension, mm	36.6 ± 5.3	36.0 ± 5.1	38.1 ± 5.8	0.136
RV dimension, mm	34.9 ± 5.6	33.4 ± 5.1	36.7 ± 6.7	0.198
Tricuspid E/A	0.92 ± 0.29	1.0 ± 0.33	1.06 ± 0.24	0.502
Tricuspid E/e′	5.7 ± 1.7	5.9 ± 2.0	5.4 ± 1.3	0.618
TAPSE, mm	21.5 ± 3.7	22.2 ± 3.5	19.1 ± 3.1	0.002
RV FAC, %	46.0 ± 5.3	47.2 ± 5.6	41.6 ± 5.5	0.001
S′, cm/s	13.4 ± 3.1	13.6 ± 3.3	12.9 ± 2.7	0.340
RV MPI	0.48 ± 0.16	0.46 ± 0.15	0.54 ± 0.19	0.045
Moderate-severe TR, *n* (%)	5 (5.6%)	3 (4.3%)	2 (10%)	0.313
PASP, mmHg	42 (27, 50)	33 (27, 43)	48 (34, 59)	0.042

*Values are mean ± SD, n (%), median (interquartile range)*.

*COVID-19, coronavirus disease 2019; DT, deceleration time; IVS, interventricular septum; LA, left atrium; LV, left ventricular; LVEDVI, left ventricular end diastolic volume index; LVESVI, left ventricular end systolic volume index; LVEF, left ventricular ejection fraction; LVM, left ventricular mass; MR, mitral regurgitation; RA, right atrium; RV, right ventricular; TAPSE, tricuspid annular plane systolic excursion; RV FAC, RV fractional area change; RV MPI, RV myocardial performance index; TR, tricuspid regurgitation; PASP, pulmonary artery systolic pressure; PW, posterior wall of left ventricle*.

### Predictors of Mortality in COVID-19 Patients With CVD

**Figure 2:**
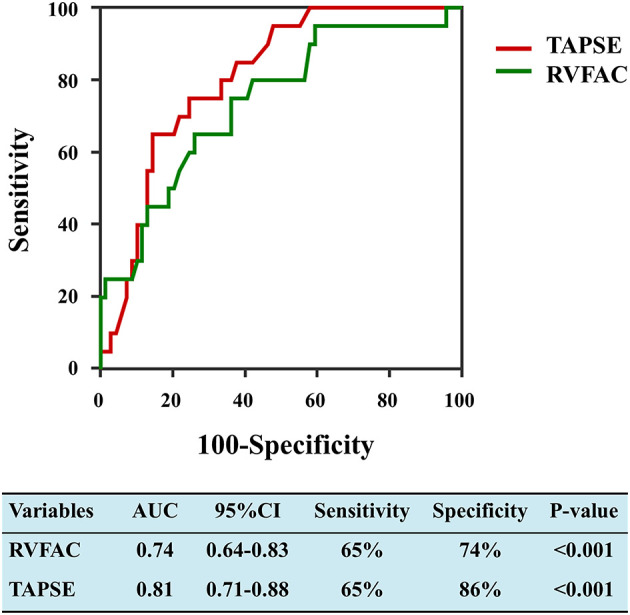
Receiver operating characteristic curves of RVFAC and TAPSE for adverse clinical outcome. RVFAC, right ventricular fractional area change; TAPSE, tricuspid annular plane systolic excursion.

LV and RV function parameters were studied by a receiver operating characteristic (ROC) analysis to evaluate the probability of mortality. RV functional indices were associated with a higher risk of mortality in COVID-19 patients with CVD (Figure 2). Area under the curve was 0.74 for RVFAC and 0.81 for TAPSE.

**Figure 3:**
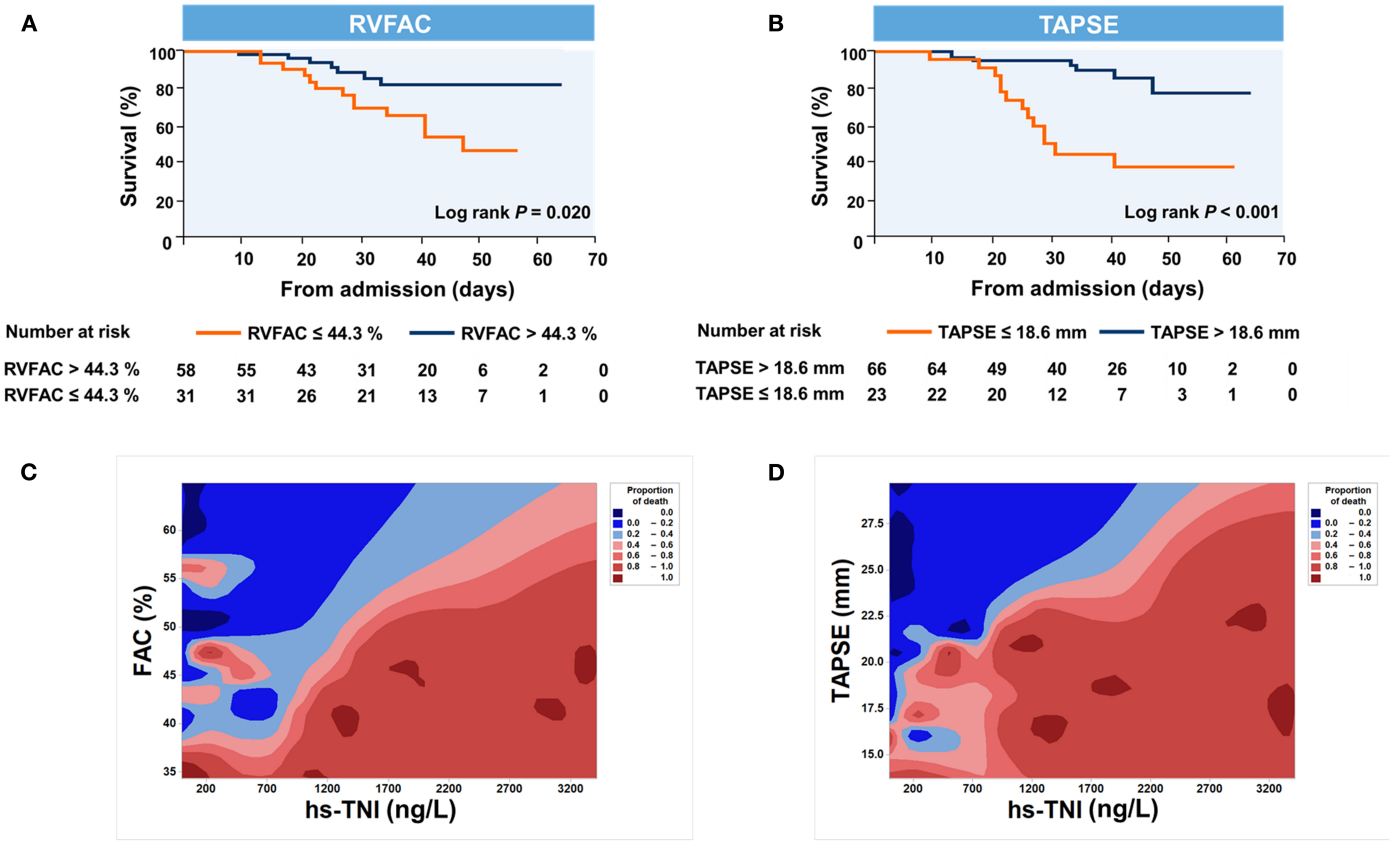
Kaplan–Meier plots and contour plots of survival probability in hospitalized COVID-19 patients with CVD. **(A,B)** Survival significantly declined with diminished TAPSE and RVFAC. **(C,D)** Decreased TAPSE and RVFAC were associated with higher mortality, which were pronounced in patients with higher levels of hs-TNI. RVFAC, right ventricular fractional area change; TAPSE, tricuspid annular plane systolic excursion; hs-TNI, high-sensitivity troponin I.

Kaplan–Meier survival curves for mortality are displayed Figures 3A,B. When stratified by cutoff values, RVFAC <44.3% or TAPSE <18.6 mm was associated with higher mortality (*p* < 0.001). To determine the relationship between levels of hs-TNI, RV function parameters, and mortality, a contour plot was performed. Our findings revealed that decreased RV function was associated with increased mortality, which was pronounced in patients with higher levels of hs-TNI (Figures 3C,D).

**Figure 4:**
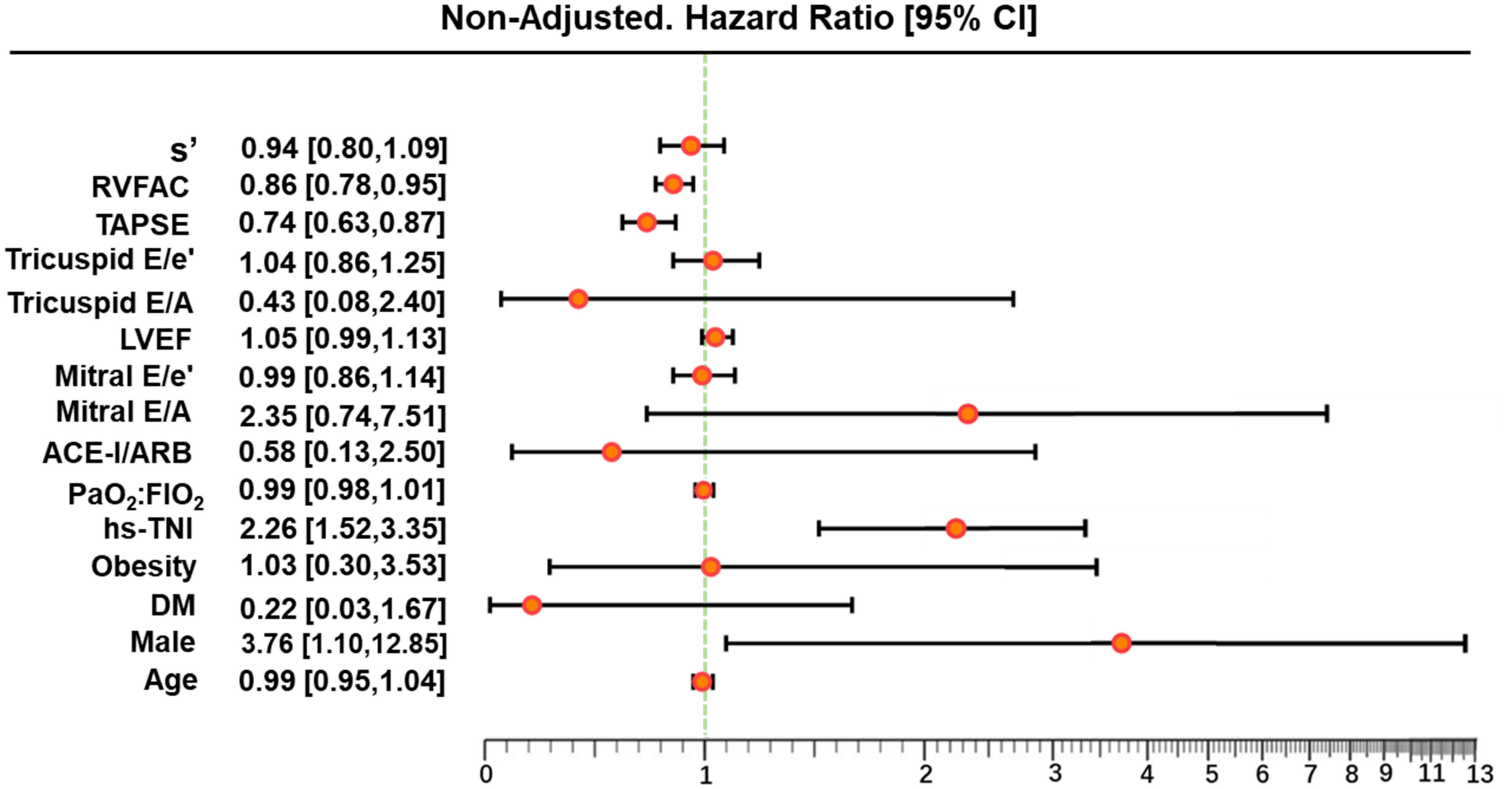
Univariate Cox regression analysis of clinical and echocardiographic parameters. Forest plot for association of clinical and echocardiographic parameters with mortality. Impact of clinical and echocardiographic indicators on mortality in COVID-19 patients with CVD. ACE-I, angiotensin-converting enzyme inhibitors; ARB, angiotensin II receptor blockers; CI, confidence interval; COVID-19, coronavirus disease 2019; CVD, cardiovascular disease; DM, diabetes mellitus; FIO_2_, fraction of inspiration oxygen; hs-TNI, hypersensitive troponin I; LVEF, left ventricular ejection fraction; RVFAC, right ventricular fractional area change; TAPSE, tricuspid annular plane systolic excursion; PaO_2_, partial pressure of oxygen.

**Figure 5:**
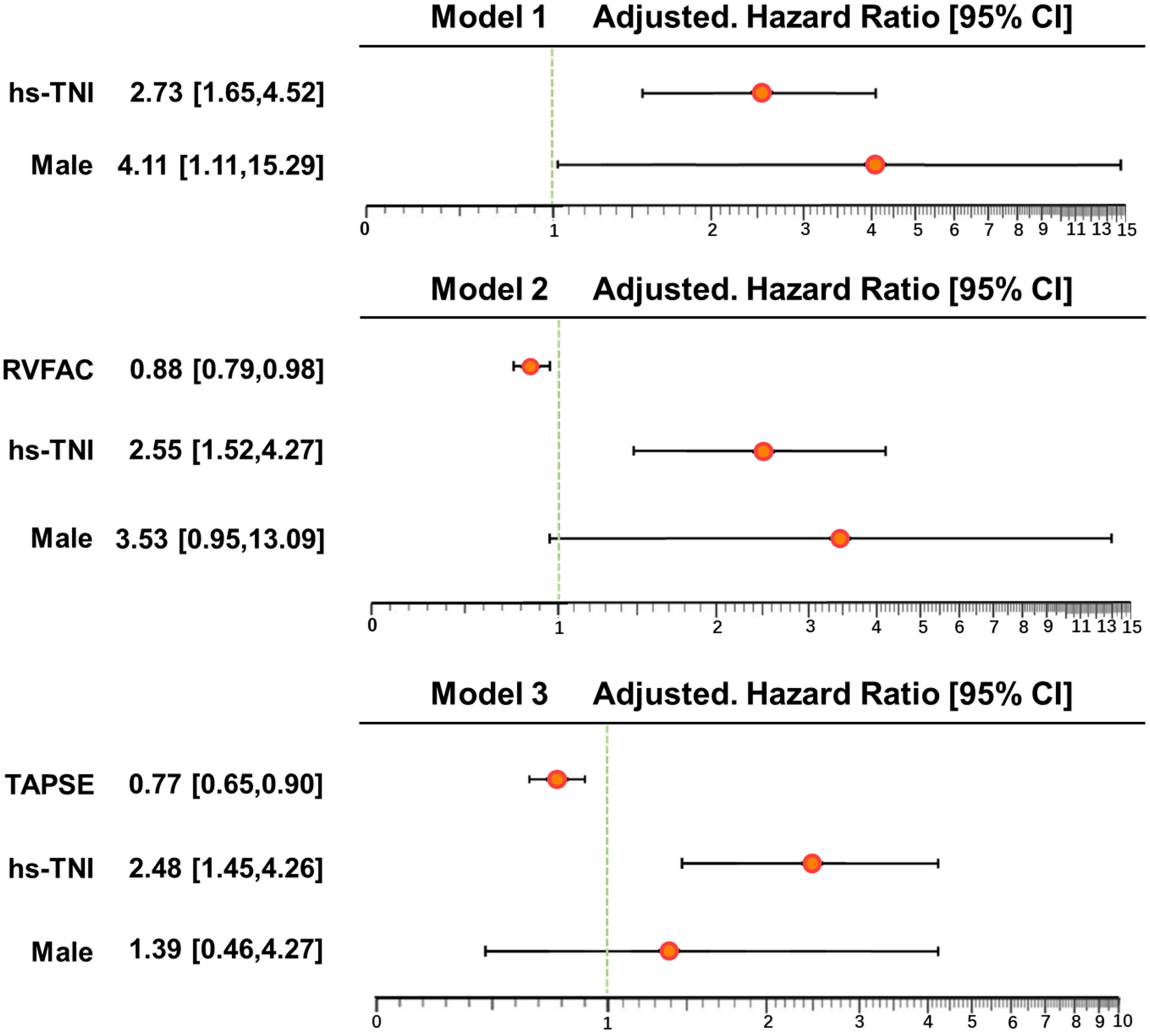
Multivariate Cox regression analysis of clinical and echocardiographic parameters. Forest plot for association of clinical and echocardiographic parameters with mortality. Impact of clinical and echocardiographic indicators on mortality in COVID-19 patients with CVD. CI, confidence interval; COVID-19, coronavirus disease 2019; CVD, cardiovascular disease; hs-TNI, hypersensitive troponin I; RVFAC, right ventricular fractional area change; TAPSE, tricuspid annular plane systolic excursion.

**Figure 6:**
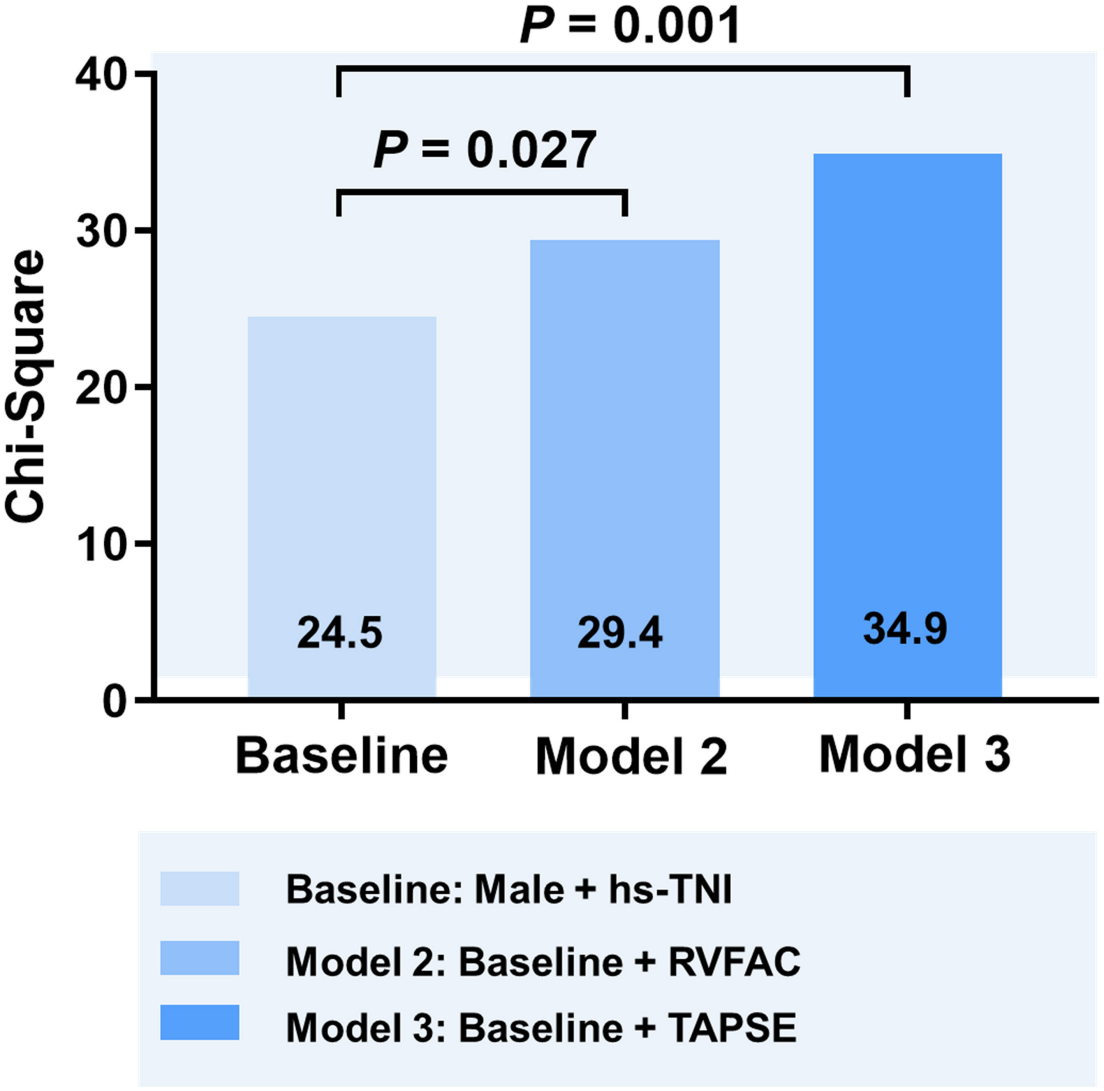
Likelihood ratio test for the incremental prognostic value of TAPSE. The incremental value of TAPSE over clinical and RVFAC for the prediction of mortality. RVFAC, right ventricular fractional area change; TAPSE, tricuspid annular plane systolic excursion; hs-TNI, high-sensitivity troponin I.

In univariate and multivariate Cox analysis, higher level of hs-TNI, TAPSE, and RVFAC were independent predictors of higher risk of mortality (Figures 4, 5). To determine the incremental prognostic value of TAPSE over RVFAC and clinical variables in COVID-19 patients with CVD, a likelihood ratio test was performed. Figure 6 compares the additional chi-square statistic value of TAPSE and RVFAC to increase predictive value for mortality. After the addition of RVFAC to the baseline model, an increase in the chi-square value was observed (chi-square difference = 4.9; *p* = 0.027). After the addition of TAPSE to the baseline model, an increased chi-square value was noted (chi-square difference = 10.4; *p* = 0.001). The incremental chi-square value of TAPSE was higher than that of RVFAC, demonstrating the additional prognostic value of TAPSE in COVID-19 patients with CVD. Moreover, the model with TAPSE (AIC = 129, C index = 0.86) was the best in predicting mortality compared with those with RVFAC (AIC = 137, C index = 0.84), and baseline model (AIC = 138, C index = 0.81).

## Discussion

To the best of our knowledge, this may be the first study describing the echocardiographic features and its prognostic value in patients with COVID-19 and CVD. COVID-19 patients with CVD displayed poorer LV diastolic and RV function than non-CVD patients. The most common cardiac abnormality in CVD patients was RV dysfunction, followed by LV diastolic dysfunction and LV systolic dysfunction. Furthermore, diminished RV function was associated with higher mortality in CVD patients, suggesting that RV measurements may be important for detecting COVID-19 patients with CVD who are at higher risk of mortality.

### COVID-19 Patients With CVD and Cardiac Injury

Consistent with a previous study, we found that COVID-19 patients with CVD had a significantly higher mortality compared to those without (11). The mechanism of poor outcomes in patients of COVID-19 with CVD remains unknown. Previous reports suggest that coronavirus viral infections may trigger cardiovascular events and exacerbate heart failure (11–13). Direct viral damage, aggravation of a systemic inflammatory response, and hypoxemia may result in cardiac injury. Our study showed that COVID-19 patients with pre-existing CVD are more susceptible to cardiac injury. Furthermore, CVD patients with hs-TNI elevation are more likely to develop severe illness. Prior studies demonstrated that cardiac injury was associated with poor clinical outcome, irrespective of a history of CVD (3, 14, 15). In the present study, CVD patients who died had a significantly higher incidence of cardiac injury compared to those who were alive. Moreover, our results further revealed that the level of hs-TNI could help identify patients at higher risk and requiring earlier or more aggressive treatment strategies.

### Cardiac Characteristics of COVID-19 Patients With CVD

Our study showed that patients with COVID-19 infection and underlying CVD had impaired LV diastolic function. This is in keeping with the study of Li et al., which demonstrated that only subclinical LV diastolic impairment was identified in patients with severe acute respiratory syndrome (16). In line with the results of Inciardi et al. (6), no difference was observed in LVEF between patients with or without CVD. Furthermore, LVEF was preserved in the majority of hospitalized CVD patients, in agreement with the results of Churchill et al., demonstrating that LVEF was normal/hyperdynamic in most patients with COVID-19 (17). Several case reports also demonstrate that the majority of patients with uncomplicated myocarditis displays normal cardiac function (17–19). In addition, diminished RV performance was the most common in patients with CVD, consistent with recent reports in unselected COVID-19 patients (20–22).

Generally, the etiology of RV dysfunction in COVID-19 infection has not been well-established. In addition to myocardial injury, it is though that the RV dysfunction may be reflective of conditions that can increase RV afterload during this viral infection, including hypoxic pulmonary vasoconstriction, hypercarbia, excessive positive end-expiratory pressure (PEEP), pneumonia, elevated left atrial pressure, or combination of all these factors (21). In a recent study of 26 symptomatic patients with COVID-19 infection (and without a history of coronary artery disease or myocarditis), Huang et al. investigated cardiac involvement using magnetic resonance imaging and found that 58% of patients displayed impaired RV function (23). Furthermore, myocardial edema and fibrosis were observed in these patients. Indeed, 30% of COVID-19 patients with CVD required mechanical ventilation at the time of echocardiogram. RV dysfunction has been demonstrated to be a complication of hypoxemic injury including ARDS and may deteriorate following mechanical ventilation due to the presence of higher PEEP causing higher RV afterload (24, 25). Importantly, we noticed that LV diastolic and RV function was further diminished in patients with CVD compared with those without. Recent evidence suggests that patients with CVD are more likely to develop severe and critical illness that may partially explain why these patients present with worsening cardiac function (3). Another possible explanation may be that SARS-CoV-2 infection might aggravate a pre-existing cardiovascular condition (26). The poorer cardiac function in COVID-19 patients with CVD may alert physicians to pay greater attention to the management of these patients.

### Prognostic Value of Echocardiographic Parameters in COVID-19 Patients With CVD

Considering that patients with COVID-19 infection and underlying CVD are more likely to have a more severe course of their illness and a poorer clinical outcome, it is imperative to identify this high-risk group for consideration of earlier or more intensive therapy. Thus far, some prognostic indicators of poor outcome, in particular elevated level of hs-TNI, have been recognized (3, 27, 28). Our current study not only verified the role of these previously reported risk prognosticators but also reported the novel and additive prognostic value of RV measurements in patients with COVID-19 infection and underlying CVD.

In our study, patients found to have reduced RV function by echocardiography were at higher risk of deterioration and death. Our results demonstrate that RV function serves as a novel imaging biomarker that predicts higher mortality in patients with COVID-19 infection and underlying CVD. These findings were consistent with our previous work showing that RV dysfunction predicted poorer outcome in unselected patients with COVID-19 (with or without CVD) (25). Similarly, in a recent study of 110 patients with COVID-19, Argulian et al. demonstrated that RV dilation was an independent predictor of in-hospital mortality (29). Importantly, our study reveals that TAPSE appears to be the best predictor of higher mortality compared with RVFAC and other clinical variables. RVFAC depends on imaging quality, resulting in relatively poor inter- and intraobserver reproducibility in subjects with suboptimal endocardial definition. In contrast, TAPSE is less dependent upon image quality, is simple to perform, and is reproducible. TAPSE is widely used on a daily basis in most echocardiographic laboratories. Considering the reduced time of exposure during echocardiographic examination in patients with COVID-19, the present study revealed the key clinical implication of TAPSE, as it can be easily obtained during bedside echocardiography. Our results highlights that the additional prognostic value of TAPSE over the other clinical parameters and RVFAC is important for risk stratification in COVID-19 patients with CVD.

### Limitations

Although our results demonstrated the presence of cardiac impairment in COVID-19 patients with underlying CVD, the time course for the development of these cardiac abnormalities remained unknown, as we did not have serial echocardiography available for these patients. Another limitation to consider is that although RV functional parameters were revealed to be important predictors of risk in this study, we only carried out the basic, commonly used measures of RV function such as TAPSE and RVFAC (30), as opposed to more advanced measures such as RV myocardial strain and RV three-dimensional imaging, which are now recommended for consideration by the ASE (31) and EACVI (32).

Finally, the main limitation of our study was that it was a single-center study, with a relatively limited sample size and a homogenous population. As a center designated to treat patients with COVID-19 in our region, our study subjects may not be representative of populations elsewhere, limiting extrapolation of our results. Future studies, involving larger sample sizes, multiple centers, and international collaboration, are needed to determine the true prognostic value of echocardiographic parameters in patients with COVID-19 infection and allow for further refinement of stratification by determinants such as sex, age, and ethnicity.

## Conclusions

Right ventricular dysfunction is more common than LV dysfunction among COVID-19 patients with underlying CVD. Importantly, RV function parameters are associated with higher mortality, suggesting that RV measurement may serve as a novel imaging biomarker for the risk stratification of patients with COVID-19 infection and underlying CVD. The study highlights the importance of bedside cardiovascular ultrasound in the assessment and prognostication of hospitalized patients with COVID-19 infection.

## Data Availability Statement

The original contributions presented in the study are included in the article/Supplementary Materials, further inquiries can be directed to the corresponding authors.

## Ethics Statement

The studies involving human participants were reviewed and approved by Tongji Medical College, Huazhong University of Science and Technology. The patients/participants provided their written informed consent to participate in this study. Written informed consent was obtained from the individual(s) for the publication of any potentially identifiable images or data included in this article.

## Author Contributions

Conception and design of the study: YL, LF, SZ, YX, JW, YY, QL, AJ, MX, and LZ. Acquisition of data: BW, LH, DZ, YoZ, and HY. Analysis and interpretation of data: CW, HL, WS, YaZ, ML, YC, and LC. Drafting the article: YL, LF, SZ, and YX. Revising the article: YL, LF, SZ, YX, LZ, and MX. Final approval of the article: SZ, YX, LY, and LZ. All authors listed have made a substantial, direct and intellectual contribution to the work, and approved it for publication.

## Conflict of Interest

The authors declare that the research was conducted in the absence of any commercial or financial relationships that could be construed as a potential conflict of interest.
