# Supplementary material for: Echocardiographic Characteristics and Outcome in Patients With COVID-19 Infection and Underlying Cardiovascular Disease
Source: Front Cardiovasc Med. 2021 Mar 16;8:642973. doi: 10.3389/fcvm.2021.642973 (PMC8008078; doi:10.3389/fcvm.2021.642973)
Supplement: Supplementary file 1 [file Data_Sheet_1.docx]

| **Supplemental Table 1. Clinical and Echocardiographic Characteristics of COVID-19 Patients with CVD According to Mechanical Ventilation Therapy** | | | |
| --- | --- | --- | --- |
| Variables | With MV (n=27) | Without MV (n=62) | *P* Value |
| Age, years | 67±10 | 66±11 | 0.752 |
| Male, n (%) | 20(74.1%) | 31(50.0%) | 0.029 |
| Body mass index, kg/m2 | 23.1±2.9 | 23.6±2.8 | 0.421 |
| Heart rate, beats/min | 90±16 | 88±16 | 0.517 |
| Respiratory rate, times/min | 26±7 | 25±6 | 0.467 |
| Systolic arterial pressure, mm Hg | 133±16 | 140±18 | 0.096 |
| Diastolic arterial pressure, mm Hg | 79±12 | 83±13 | 0.185 |
| hs-TNI, ng/L | 56.2(29.5,131.3) | 4.2(2.4,16.3) | < 0.001 |
| CK-MB, U/L | 20(13,30) | 10(7,15) | 0.121 |
| BNP, pg/ml | 86.9(37.6,193.9) | 73.2(34.0,144.2) | 0.72 |
| CRP, mg/L | 80.6(33.2,128.0) | 13.1(3.7,52.0) | < 0.001 |
| PCT, ng/ml | 0.19(0.11,0.36) | 0.07(0.05,0.15) | 0.05 |
| IL-6, pg/ml | 27.0(12.0,88.0) | 4.5(3.0,13.5) | 0.246 |
| D-dimer, mg/L | 1.2(0.4,2.2) | 1.5(0.4,2.6) | 0.423 |
| **Left heart** |  |  |  |
| LA, mm | 38.3±6.7 | 36.1±5.4 | 0.118 |
| LV, mm | 44.7±5.4 | 46.2±4.8 | 0.235 |
| IVS, mm | 9.6±1.4 | 9.9±1.3 | 0.36 |
| PW, mm | 9.4±1.3 | 9.1±1.4 | 0.299 |
| LVMI, g/m^2^ | 85.6±29.6 | 89.7±20.3 | 0.485 |
| Mitral E/A | 0.99±0.41 | 0.82±0.28 | 0.059 |
| Mitral E/e' | 10.1±3.0 | 9.5±3.6 | 0.435 |
| LVEDVI, mL/m^2^ | 53.7(45.3,64.1) | 52.4(43.2,63.8) | 0.139 |
| LVESVI, mL/m^2^ | 23.4(11.4,29.8) | 21.6(16.5,28.4) | 0.141 |
| LVEF, % | 64.2±6.8 | 61.7±8.8 | 0.236 |
| **Right heart** |  |  |  |
| RA, mm | 38.2±5.7 | 35.8±5.0 | 0.065 |
| RV, mm | 36.5±5.7 | 34.2±5.5 | 0.08 |
| Tricuspid E/A | 0.93±0.35 | 0.92±0.27 | 0.813 |
| Tricuspid E/e' | 5.8±2.5 | 4.7±2.2 | 0.049 |
| TAPSE, mm | 19.2±2.9 | 22.5±3.5 | < 0.001 |
| RV FAC, % | 43.5±5.3 | 47.0±6.0 | 0.03 |
| S’, cm/s | 13.6±3.1 | 13.4±3.2 | 0.845 |
| RV MPI | 0.53±0.21 | 0.46±0.13 | 0.083 |
| PASP, mmHg | 50(43,58) | 32(26,38) | < 0.001 |
| Data are mean ± SD, n (%), median (IQR). SD, standard deviation; IQR, interquartile range. BNP, B-type natriuretic peptide; CK-MB, creatine kinase muscle-brain; CRP, C-reactive protein; hs-TNI, high-sensitivity troponin I; IL-6, interleukin-6; PCT, procalcitonin; COVID-19, coronavirus disease 2019; CVD, cardiovascular disease; IVS, interventricular septum; LA, left atrium; LV, left ventricular; LVEDVI, left ventricular end diastolic volume index; LVEF, left ventricular ejection fraction; LVESVI, left ventricular end systolic volume index; LVM, left ventricular mass; PW, posterior wall of left ventricle; RA, right atrium; RV, right ventricular; RVFAC, right ventricular fractional area change; RV MPI, RV myocardial performance index; TAPSE, tricuspid annular plane systolic excursion; PASP, pulmonary artery systolic pressure. | | | |

| **Supplemental Table 2. Correlations Between hs-TNI and Echocardiographic Parameters in Patients with COVID-19.** | | |
| --- | --- | --- |
|  | hs-TNI, ng/L | |
| Variables | r | *P* Value |
| **Left heart** |  |  |
| LA dimension, mm | 0.30 | 0.001 |
| LV dimension, mm | 0.04 | 0.702 |
| Mitral E/A | 0.02 | 0.846 |
| Mitral E/e' | 0.28 | 0.002 |
| LVEDVI, mL/m2 | - 0.06 | 0.547 |
| LVESVI, mL/m2 | - 0.06 | 0.558 |
| LVEF, % | 0.08 | 0.409 |
| **Right heart** |  |  |
| RA dimension, mm | 0.27 | 0.002 |
| RV dimension, mm | 0.22 | 0.012 |
| Tricuspid E/A | - 0.21 | 0.033 |
| Tricuspid E/e' | - 0.22 | 0.340 |
| TAPSE, mm | - 0.29 | 0.001 |
| RVFAC, % | - 0.27 | 0.005 |
| S’, cm/s | - 0.16 | 0.072 |
| RV MPI | 0.27 | 0.003 |
| COVID-19, coronavirus disease 2019; hs-TNI, high-sensitivity troponin I; LA, left atrium; LV, left ventricular; LVEDVI, left ventricular end diastolic volume index; LVESVI, left ventricular end systolic volume index; LVEF, left ventricular ejection fraction; RA, right atrium; RV, right ventricular; TAPSE, tricuspid annular plane systolic excursion; RV FAC, RV fractional area change; RV MPI, RV myocardial performance index. | | |

| **Supplemental Table 3. Clinical Characteristics of COVID-19 Patients with CVD Stratified by Vital Status** | | | | |
| --- | --- | --- | --- | --- |
| Variables | With CVD (n=89) | Survivors (n=69) | Non-survivors (n=20) | *P* Value |
| **Clinical characteristics** |  |  |  |  |
| Age, years | 66±11 | 66 ±10 | 66±11 | 0.929 |
| Male, n (%) | 51(57.3%) | 34(49.3%) | 17(85.0%) | 0.004 |
| Body mass index, kg/m^2^ | 24.0±3.0 | 24.1±2.9 | 23.4±3.4 | 0.395 |
| Heart rate, beats/min | 89±16 | 88±15 | 91±18 | 0.488 |
| Respiratory rate, breaths/min | 25±6 | 24±6 | 27±8 | 0.303 |
| Systolic arterial pressure, mm Hg | 138±17 | 139±18 | 134±17 | 0.238 |
| Diastolic arterial pressure, mm Hg | 82±13 | 83±13 | 79±13 | 0.187 |
| Smoker, n (%) | 11(12.4%) | 7(10.1%) | 4(20.0%) | 0.238 |
| **Comorbidities** |  |  |  |  |
| Hypertension, n (%) | 70(78.7%) | 55(79.7%) | 15(75.0%) | 0.651 |
| Diabetes, n (%) | 17(19.1%) | 16(23.2%) | 1(5.0%) | 0.058 |
| Obesity, n (%) | 15(16.9%) | 12(17.4%) | 3(15.0%) | 0.801 |
| COPD, n (%) | 6(6.7%) | 3(4.3%) | 3(15.0%) | 0.124 |
| Coronary artery disease, n (%) | 26(29.2%) | 23(33.3%) | 3(15.0%) | 0.112 |
| Heart failure, n (%) | 4(4.5%) | 0(0%) | 4(20.0%) | 0.002 |
| Arrhythmia, n (%) | 6(6.7%) | 5(7.2%) | 1(5.0%) | 1.000 |
| Chronic kidney disease, n (%) | 2(2.2%) | 2(2.9%) | 0(0%) | 1.000 |
| Chronic liver disease, n (%) | 2(2.2%) | 2(2.9%) | 0(1%) | 1.000 |
| Malignancy, n (%) | 3(3.4%) | 2(2.10%) | 1(5.0%) | 0.539 |
| **Laboratory findings** |  |  |  |  |
| Lymphocyte count, × 10⁹/L | 0.9(0.5,1.2) | 1.1(0.7,1.4) | 0.4(0.3,0.6) | <0.001 |
| D-dimer, mg/L | 1.5(0.4,2.4) | 0.9(0.3,2.2) | 1.8(1.3,3.1) | 0.075 |
| PT, s | 13.4(12.6,15.2) | 13.2(12.4,14.0) | 15.9(15.1,18.1) | <0.001 |
| APTT, s | 38.0(33.1,45.6) | 36.8(32.5,40.4) | 45.7(39.4,52.9) | <0.001 |
| CK-MB, U/L | 12(8,25) | 11(8,18) | 21(14,31) | 0.003 |
| hs-TNI, ng/L | 10.6(3.3,53.7) | 5.2(2.25.4) | 105.0(30.4,615.8) | <0.001 |
| BNP, pg/ml | 85.3(34.6,162.5) | 78.5(34.6,140.9) | 129.5(34.6,641.7) | 0.284 |
| CRP, mg/L | 27.5(7.1,75.4) | 18.2(3.8,52.9) | 81.7(51.9,127.6) | <0.001 |
| PCT, ng/ml | 0.10(0.05,0.20) | 0.08(0.05,0.14) | 0.27(0.11,0.41) | <0.001 |
| IL-6, pg/ml | 8.9(3.5,21.6) | 6.1(3.3,16.5) | 153.9(97.4,494.9) | <0.001 |
| PaO_2_:FIO_2_, mmHg | 212.1(140.6,241.5) | 213.0(149.5,250.8) | 151.0(110.0,182.0) | 0.133 |
| **Treatments** |  |  |  |  |
| Antiviral therapy, n (%) | 86(96.6%) | 66(95.7%) | 20(100.0%) | 0.343 |
| Antibiotic therapy, n (%) | 73(82.0%) | 54(78.3%) | 19(95.0%) | 0.086 |
| Glucocorticoid therapy, n (%) | 36(40.4%) | 18(26.1%) | 18(90.0%) | <0.001 |
| Intravenous immune globulin, n (%) | 37(41.6%) | 21(30.4%) | 16(80.0%) | <0.001 |
| Anticoagulant therapy, n (%) | 52(58.4%) | 34(49.3%) | 18(90.0%) | 0.001 |
| Diuretics, n (%) | 32(36.0%) | 16(24.8%) | 16(80.0%) | <0.001 |
| Beta-blockers, n (%) | 28(31.5%) | 21(30.4%) | 7(35.0%) | 0.699 |
| Calcium channel blockers, n (%) | 43(48.3%) | 36(52.2%) | 7(35.0%) | 0.176 |
| ACE-I/ARB, n (%) | 15(16.9%) | 13(18.8%) | 2(10.0%) | 0.505 |
| Oxygen therapy, n (%) | 83(93.3%) | 63(91.3%) | 20(100.0%) | 0.034 |
| High-flow oxygen, n (%) | 61(68.5%) | 41(59.4%) | 20(100.0%) | 0.001 |
| Mechanical ventilation, n (%) | 27(30.3%) | 10(14.5%) | 17(85.0%) | <0.001 |
| IMV, n (%) | 19(21.3%) | 6(8.7%) | 13(65.0%) | <0.001 |
| NIMV, n (%) | 8(9.0%) | 4(5.8%) | 4(20.0%) | 0.072 |
| ICU admission, n (%) | 20(22.5%) | 6(8.7%) | 14(70.0%) | <0.001 |
| **Complications** |  |  |  |  |
| Acute kidney injury, n (%) | 12(13.5%) | 5(7.2%) | 7(35.0%) | 0.004 |
| ARDS, n (%) | 47(52.8%) | 28(40.6%) | 19(95.0%) | <0.001 |
| Acute heart injury, n (%) | 35(39.3%) | 15(21.7%) | 20(100.0%) | <0.001 |
| Coagulation dysfunction, n (%) | 19(21.3%) | 9(13.0%) | 10(50.0%) | 0.001 |
| DVT, n (%) | 42(47.2%) | 26(37.7%) | 16(80.0%) | 0.001 |
| Shock, n (%) | 1(1.1%) | 0(0%) | 1(5.0%) | 0.225 |
| **Prognosis** |  |  |  |  |
| Discharge, n (%) | 69(77.5%) | 69(100.0%) | 0(0%) | <0.001 |
| Death, n (%) | 20(22.5%) | 0(0%) | 20(100.0%) | <0.001 |

Values are mean ± SD, n (%), median (interquartile range). ACE-I, angiotensin-converting enzyme inhibitors; APTT, activated partial thromboplastin time; ARB, angiotensin II receptor blockers; ARDS, acute respiratory distress syndrome; BNP, B-type natriuretic peptide; CK-MB, creatine kinase muscle-brain; COVID-19, coronavirus disease 2019; COPD, chronic obstructive pulmonary disease; CRP, C-reactive protein; DVT, deep vein thrombosis; FIO_2_, fraction of inspiration oxygen; HF, heart failure; hs-TNI, high-sensitivity troponin I; ICU, intensive care unit; IL-6, interleukin-6; IMV, invasive mechanical ventilation; NIMV, non-invasive mechanical ventilation; PCT, procalcitonin; PT, prothrombin time; PaO_2_, partial pressure of oxygen.
